# 

**Published:** 1911-03-04

**Authors:** 


Telegraphic Address: Ospedale, London.

THE MODERN MEDICAL AND INSTITUTIONAL JOURNAL.

Telephone Number: 2734 Gerrard.

NEW SERIES. No. 213, Vol. VIII. No. 1285, Vol. XLIX.

SATURDAY, MARCH 4TH, 1911.

PRICE THREEPENCE

